# Inhibition of SPATS2 Suppresses Proliferation and Invasion of Hepatocellular Carcinoma Cells through TRIM44-STAT3 Signaling Pathway

**DOI:** 10.7150/jca.47526

**Published:** 2021-01-01

**Authors:** Lirong Chen, Chenhe Yi, Wenshuai Li, Yujen Tseng, Jun Zhang, Jie Liu

**Affiliations:** 1 Department of Digestive Diseases, Huashan Hospital, Fudan University, Shanghai, PR China.; 2 Department of General Surgery, Huashan Hospital, Fudan University, Shanghai, PR China.

**Keywords:** SPATS2, TRIM44, STAT3, Hepatocellular carcinoma

## Abstract

Hepatocellular carcinoma (HCC) is a major global health burden and its treatment options are limited. Spermatogenesis associated serine rich 2(SPATS2), a recent defined oncogene, was found to be a prognostic biomarker in HCC. However, the explicit mechanism underlying SPATS2 was urged to be elucidated. In vitro, knockdown of SPATS2 hampered the proliferation, invasion and migration of HCC cells. Moreover, phosphorylation of signal transducer and activator of transcription 3 (STAT3) and its downstream oncogenes were dramatically suppressed by SPATS2 knockdown. In addition, tripartite motif containing 44 (TRIM44) was found to be positively associated with SPATS2 in TCGA and declined after SPATS2 knockdown in HCC cells. Overexpression of TRIM44 rescued the effect of SPATS2 silencing on p-STAT3 and its downstream oncogenes. In vivo, SPATS2 silencing was confirmed to impede HCC tumor development in nude mice. In our own cohort containing 112 HCC patients, high SPATS2 protein level is indicative of an unfavorable clinicopathological feature and poor prognosis and could serve as an independent risk factor. Collectively, the present study is the first to propose the mechanism of significance of SPATS2-TRIM44-p-STAT3 in HCC and provide a new theoretical basis for targeted therapy.

## Introduction

Hepatocellular carcinoma (HCC) predominantly accounts for 80%-90% of liver cancer, which is estimated to be the second leading killer of cancer-associated death in the world^1^. With approximately over 850,000 new cases annually, the epidemic tendency of HCC is still on the rise^2^. Although risk factors of HCC have been well established as viral hepatitis B and C, cirrhosis, fatty liver disease, diabetes, aflatoxin, and aristolochic acid, the diagnosis, treatment and prognosis remain as daunting tasks^3^. Identifying novel molecules will be helpful in reducing this heavy health burden.

Spermatogenesis associated serine rich 2 (SPATS2) locates in cytoplasm and is expressed in 217 organs, highest in testis and developmentally regulated during spermatogenesis^4^. In addition, SPATS2 is also involved in cancer research. Previous study identified SPATS2 as an accurate diagnosis biomarker of squamous cell carcinoma^5^. It was demonstrated that SPATS2 was a differentially-enriched gene in poor-prognosis prostate cancer^6^. SPATS2 also promoted survival of colorectal cancer cells through pro-proliferative and anti-apoptotic effect^7^. Recently, SPATS2 expression was reported to be a diagnostic and prognostic biomarker in liver cancer. However, to date, little attention has been paid to the mechanism between SPATS2 and HCC.

Tripartite motif containing 44 (TRIM44) belongs to TRIM families containing a zinc finger ubiquitin protease domain in the N-terminal domains^8^. Accumulating studies suggest that TRIM44 functions as stimulatory and cancerogenic factor in multiple cancers. TRIM44 facilitated proliferation, migration, and invasion in colorectal cancer, melanoma, esophageal cancer and hepatocellular carcinoma^9^-^12^. It was reported that TRIM44 enhanced phosphorylation of signal transducer and activator of transcription 3 (STAT3)^10^. As canonical signal transducer, STAT3 has a crucial role in tumor genesis and progress through regulating target genes^13^. Many studies suggested that p-STAT3, as the activated form, promoted cancer-enabling proliferation, survival, invasion and metastasis, angiogenesis, replicative potential and insensitivity to anti-growth signals^14^. While p-STAT3 has proved to be a difficult drug target, identifying molecules targeting p-STAT3 displayed great value in anti-tumor therapy.

The present study provides the first investigation into the significant mechanism of SPATS2 in HCC, which helps clarify the meaning of SPATS2-TRIM44-STAT3 axis in HCC diagnosis and treatment.

## Materials and Methods

### Patients, specimens and tissue microarrays

Cancer tissue specimens and paraffin sections of adjacent tissues were collected from 112 patients with HCC who received surgical treatment at Huashan Hospital, Fudan University. Diagnosis of HCC for all patients was pathologically confirmed. The usage of patients' tissue specimens was approved by the Ethics Committee of Huashan Hospital, Fudan University and informed consents were acquired from all patients. The work described has been carried out in accordance with the principles of Declaration of Helsinki for experiments involving humans.

### Animals

All experimental procedures involving animals were approved by the Animal Care and Use Committee of Fudan University, China. MHCC-97H cells (1×10^7^) stably transfected with shSPATS2 or control vectors were subcutaneously injected into nude mice (BALB/c nu/nu, 4 weeks old) (Lingchang Biotech, China). Tumors were removed after 4 weeks. All animal experiments followed the Interdisciplinary Principles and Guidelines for the Use of Animals in Research, Testing, and Education by the New York Academy of Sciences, Ad Hoc Animal Research Committee.

### The Cancer Genome Atlas Database

The Cancer Genome Atlas (TCGA) database was used to analyze the expression of SPATS2 in patient HCC tissues. LIHC patients were divided into low- and high-expression groups and the values of SPATS2 related to prognostic analysis were examined.

### Cell culture and plasmid transfection

L02 and six HCC cell lines of huh-7, Hep-3B, PLC, Hep-G2, MHCC-97H and HCC-LM3 were used in the subsequent experimental investigations. Cells were cultured in Dulbecco's modified Eagle's medium (DMEM) with 10% fetal bovine serum (Gibco, USA) and 100 mg/mL penicillin-streptomycin in a humidified atmosphere containing 5% CO_2_ at 37 °C. Then SPATS2 shRNA and TRIM44 overexpression plasmid were constructed. HEK293T cells were used for lentivirus production. Both MHCC-97H and HCC-LM3 cells with stable SPATS2 knockdowns and TRIM44 overexpression were generated by lentiviral infection and then followed by selection with 2 μg/mL puromycin for 2 weeks. The sequences of the primers of SPATS2 shRNA were listed as follows: sh-SPATS2-1-F CCGGGCACTTTGTTAGTGAACGTAACTCGAGTTACGTTCACTAACAAAGTGCTTTTTTG, sh-SPATS2-1-R AATTCAAAAAGCACTTTGTTAGTGAACGTAACTCGAGTTACGTTCACTAACAAAGTGCT; sh-SPATS2-2-F CCGGGCACGGTATCGAGTTGTAGTTCTCGAGAACTACAACTCGATACCGTGCTTTTTTG; sh-SPATS2-2-R AATTCAAAAAGCACGGTATCGAGTTGTAGTTCTCGAGAACTACAACTCGATACCGTGCT.

### Western blotting

RIPA (Weiao, China) was used to lyse harvested cells. After electrophoresis and transfer, the PVDF membranes were incubated and shake overnight with primary antibodies at 4°C. Then, the imprints were washed with TBST for 3 times and cultured with incubated with an HRP-conjugated secondaey antibody (diluted at 1:5000) at room temperature for 1 hour. The proteins were visualized using a Immobilon^TM^ Western Chemiluminescent HRP substrate (Millipore, USA). The primary antibodies used were: Alpha Tubulin (Proteintech, 66031-1-lg), BCL2 (Proteintech,60178-1-lg), HIF-1α (Proteintech, 20962-1-AP), MMP9 (Proteintech, 10375-2-AP), PIM1 (Cell Signaling Technology, 3247T), STAT3 (Cell Signaling Technology, 9139), p-STAT3 (Cell Signaling Technology, 9145), TRIM44 (Proteintech, 11511-1-AP) and SPATS2 (Santa Crus, sc-390306).

### RNA extraction and real-time PCR assay

Total RNA was extracted from cultured cells using Trizol (Invitrogen, California, USA), and reversely transcribed to cDNA. Next, the q-PCR assays were performed using TB Green (Takara, Japan) with a q-PCR machine (Applied Biosystems, USA) according to manufacturer's protocol. The relative expression of mRNA was calculated by 2^-△△Ct^ method. The sequences of the primers were listed as follows: β-Tubulin-F CTGCTCATCAGCAAAGTGCG, β-Tubulin-R TGCGGAAGCAGATGTCGTAG; SPATS2-F AAGAGAAGATAAATGCGGTACG, SPATS2-R TACTTCACTGGCACTACCTTCC; STAT3-F ATCACGCCTTCTACAGACTGC, STAT3-R CATCCTGGAGATTCTCTACCACT; TRIM44-F AGTAACTCGGGACCAAATGAAGA, TRIM44-R CATGTGGGATTGGATGTCTGC; BCL2-F GGTGGGGTCATGTGTGTGG, BCL2-R CGGTTCAGGTACTCAGTCATCC; MMP9-F TGTACCGCTATGGTTACACTCG, MMP9-R GGCAGGGACAGTTGCTTCT; HIF-1α-F GAACGTCGAAAAGAAAAGTCTCG, HIF-1α-R CCTTATCAAGATGCGAACTCACA; PIM1-F GAGAAGGACCGGATTTCCGAC, PIM1-R CAGTCCAGGAGCCTAATGACG.

### Colony formation assay

Cells were plated in 6-well plates at a density of 1000 cells per well. The DMEM containing 10% FBS was changed every 3 days. After culture for 2 weeks, colonies were fixed with 4% methanol and stained with Jimsa dye for 20 min. Clones were counted directly with the naked eye or under a microscope (low magnification).

### Cell proliferation assay

Cell proliferation was measured with the Cell Counting Kit-8 (CCK-8) (Dojindo, China) according to the manufacturer's recommendation. Cells were plated at a density of 2×10^3^ cells per well in 96-well plates and incubated at 37°C. Proliferation rates were determined at 24, 48, 72 and 96h, and the absorbance at 450nm was performed using the CloneSelect Imager System (Genetix).

### Wound healing assay

Cells were seeded in 6-well plates and cultured until confluent. A 200μl pipette tip was used to make a straight scratch simulating a wound. Then, cells were cultured in DMEM containing 10% FBS. The wound width was observed under a microscope (Leica, UK) and measured by ImageJ software at 0, 24, 48 and 72h after scratching to calculate the rate of closure of the open wounds.

### Transwell assay

Invasion assays were performed using transwell chambers (24-well plate, 8 μm pores; Corning, US) coated with Marigel (Corning, US). In brief, 1×10^5^ cells were suspended in serum-free medium and seeded in the upper chambers, then incubated at 37°C for 36h. The lower chambers were filled with 600 μl DMEM containing 20% FBS as chemoattractant. Then, the non-migrated cells on the upper surface of the filters were removed using cotton wool swabs. The migrated cells on the lower side of the membrane were fixed in 4% methanol and stained with Jimsa dye, washed with PBS for 3 times, air-dried, then counted under a microscope (Leica, UK).

### Immunohistochemistry

The sections were incubated with 3% hydrogen peroxide for 10 minutes at room temperature to block endogenous peroxidase activity. To block non-specific binding of antibodies, slides were incubated with 5% normal goat serum in PBS containing 0.1% Tween 20 (PBST) for 1 hour at room temperature. Slides were then incubated with primary antibodies against SPATS2 at 4 ° C overnight. After washing, slides were incubated with HRP-labeled secondary antibody. Before counterstaining with hematoxylin, signals were developed with DAB solution. The intensity of immunohistochemical staining was analyzed by ImageJ software.

### Statistical analysis

Statistical analyses were performed using SPSS V20 and GraphPad Prism V8 statistical software. All data were presented as mean ± SEM based on at least three independent experiments. The experimental data were analyzed by Student's t-test (two-sided). Kaplan-Meier method was used to evaluate the cumulative survival, and the significance of the differences was using the log-rank test. The correlation of SPATS2 expression and clinical characteristics were analyzed by Pearson χ^2^. Cox multivariate regression analysis was used to determine the independent prognostic factors of HCC. P-value<0.05 was considered statistically significant.

## Results

### Knockdown of SPATS2 affects proliferation, migration and invasion of HCC cells

**Figure 1:**
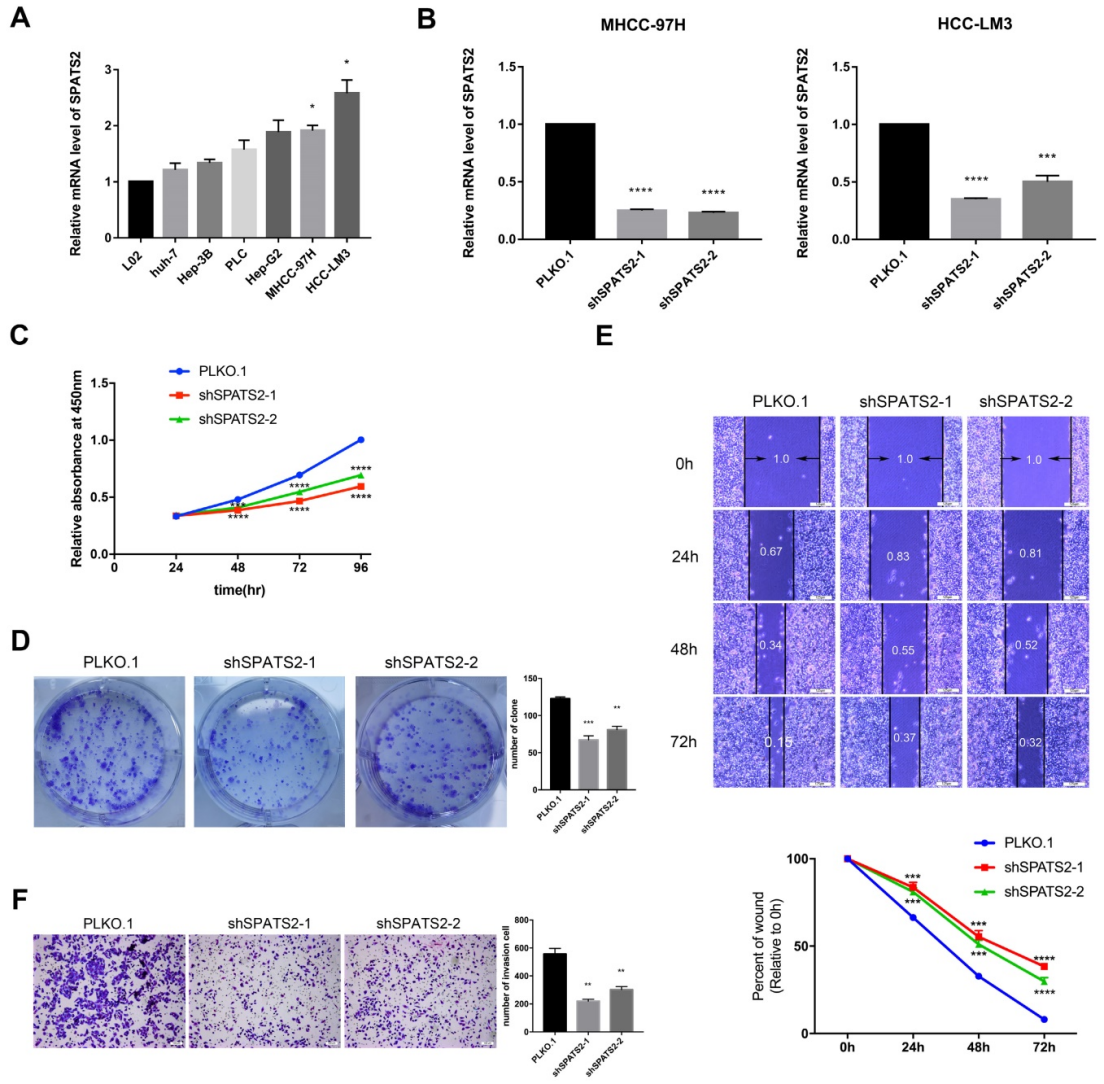
Knockdown of SPATS2 suppresses proliferation, invasion and migration of HCC cell lines. (A) SPATS2 mRNA expression of L02, Huh7, Hep3B, PLC, Hep-G2, MHCC-97H and HCC-LM3 was analyzed by q-PCR. (B) Knockdown efficiencies of SPATS2 in MHCC-97 and HCC-LM3 were confirmed by q-PCR. (C) Different proliferation abilities were measured by CCK-8 assay. (D)Different proliferation abilities were measured by colony formation assay. (E)Different migration abilities were measured by wound healing assay. (F)Different invasion abilities were measured by transwell assay. Each value represents the mean± SEM of three independent experiments. *P < 0.05, **P <0.01, ***P < 0.001, **** P < 0.0001, vs. the marked control.

To determine the function of SPATS2, we profiled its transcript level in a panel of HCC cell lines. The transcript level was observed to be higher in HCC cell lines than that in L02 (Figure 1A). Then, in MHCC-97H and HCC-LM3, two HCC cell lines with the highest SPATS2 expression, we designed two independent shRNAs for each cell line to silence SPATS2 with knockdown efficiencies>70% (Figure 1B). Confirmed by CCK8 assays, knockdown of SPATS2 substantially decreased the proliferation of HCC cells, which was further supported by colony formation assays (Figure 1C and D). In addition, wound healing assays showed that SPATS2 silencing inhibited migration dramatically (Figure 1E). Consistently, the number of invasive cells was less in shRNA groups, indicating damaged invasion ability, as monitored by transwell assays (Figure 1F). In summary, these phenotypes reveal that SPATS2 inhibition impedes proliferation, invasion and migration of HCC cells in vitro.

### Knockdown of SPATS2 inhibits HCC processes via TRIM44-STAT3 signaling pathway

**Figure 2:**
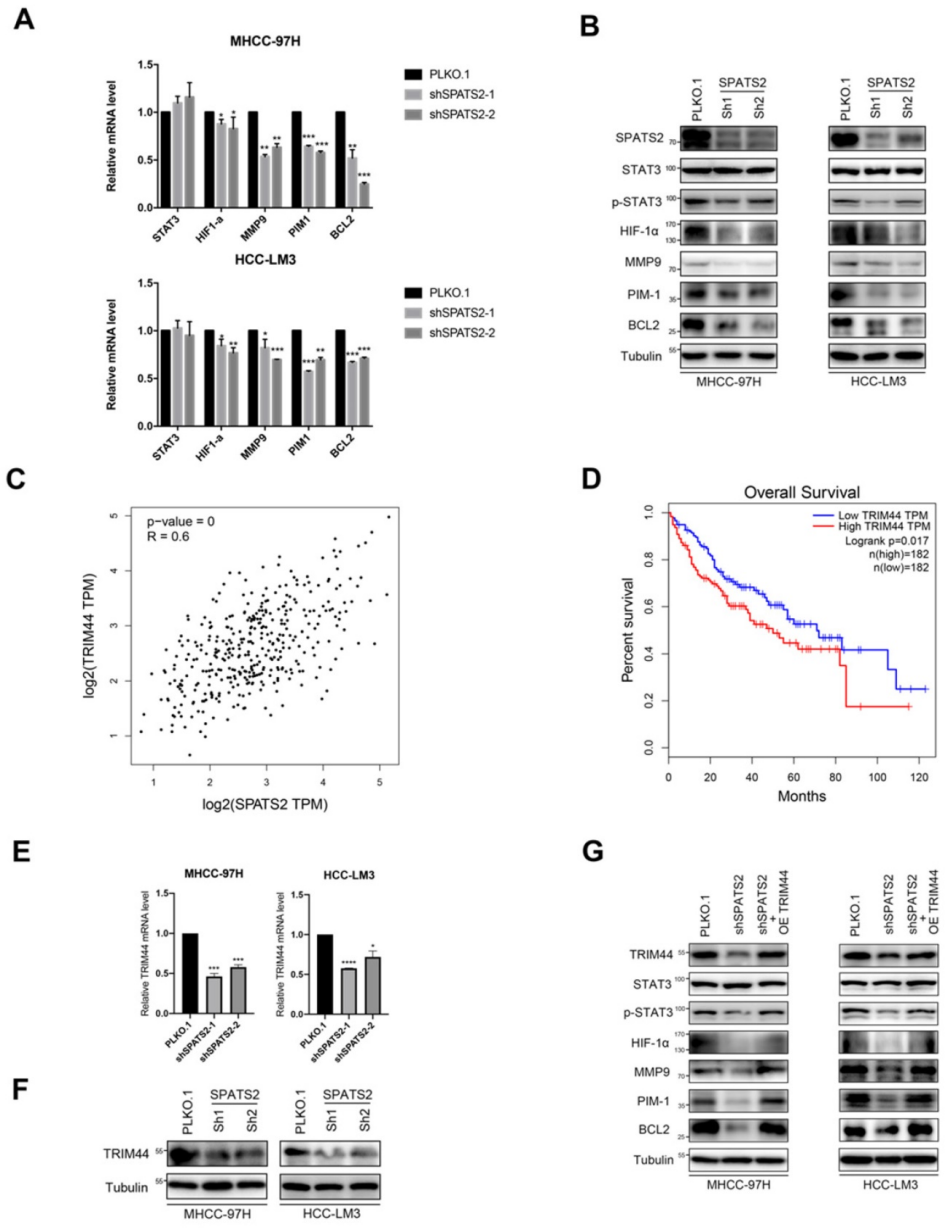
SPATS2 silencing inhibits TRIM44-STAT3 axis in HCC cells. (A) q-PCR for STAT3 and its downstream target genes in MHCC-97H and HCC-LM3 cells after SPATS2 silencing. (B) Western blotting for SPATS2, STAT3, p-STAT3 and the downstream proteins in MHCC-97H and HCC-LM3 cells after SPATS2 silencing. (C) Pearson Correlation Coefficient analysis between TRIM44 and SPATS2. (D) Kaplan-Meier curves for overall survival of TRIM44 in LIHC TCGA database. (E) q-PCR and (F)Western blotting for TRIM44 in MHCC-97H and HCC-LM3 cells after SPATS2 silencing. (G)Western blotting for TRIM44, STAT3, p-STAT3 and the downstream proteins after TRIM44 overexpression on the basis of SPATS2 knockdown. The data were shown as the mean ± SEM and representative of at least three independent experiments. *P < 0.05, **P <0.01, ***P < 0.001, **** P < 0.0001, vs. the marked control.

Since SPATS2 has been implicated in cancer-related processes, to further explore the underlying mechanism, we focused on STAT3 and its downstream pathways. Q-PCR was used to reveal that the transcriptional levels of STAT3-supporting cancer cell-intrinsic hallmarks, including BCL2, MMP9, HIF-1α and PIM1, were all decreased with SPATS2 knockdown in both MHCC-97H and HCC-LM3 cell lines (Figure 2A). Consistent with mRNA results, protein levels of these hallmarks were also reduced in all shSPATS2 groups (Figure 2B). Interestingly, STAT3 expression was neither observed in mRNA level nor protein level, while p-STAT3 declined after SPATS2 knockdown in Western blotting (Figure 2A and B). These data suggest that p-STAT3 is a pivotal mechanism for the effect of SPATS2 in HCC.

As reported, SPATS2 functioned as RNA-binding proteins (RBPs), which determined RNA fate from synthesis to decay. We searched Gene section in NCBI's databases for candidate interacting genes of SPATS2. Among these genes, we found a strong positive linear correlation between TRIM44 and SPATS2 by Pearson correlation coefficient (Figure 2C). Kaplan-Meier analysis also suggested that the higher the expression of TRIM44 in HCC, the worse the overall survival (Figure 2D). Next, reduced TRIM44 expression in mRNA and protein level was investigated in both MHCC-97H and HCC-LM3 cell lines with SPATS2 knockdown (Figure 2E and F). Subsequently, Western blotting found that decreased protein levels of p-STAT3, BCL2, MMP9, HIF-1α and PIM1 was all rescued after TRIM44 overexpression on the basis of SPATS2 knockdown (Figure 2G). In conclusion, SPATS2 inhibition obstructs HCC by regulating the cancer cell-intrinsic hallmarks through TRIM44-STAT3 axis.

### Inhibition of SPATS2 suppresses xenograft tumor development in vivo

**Figure 3:**
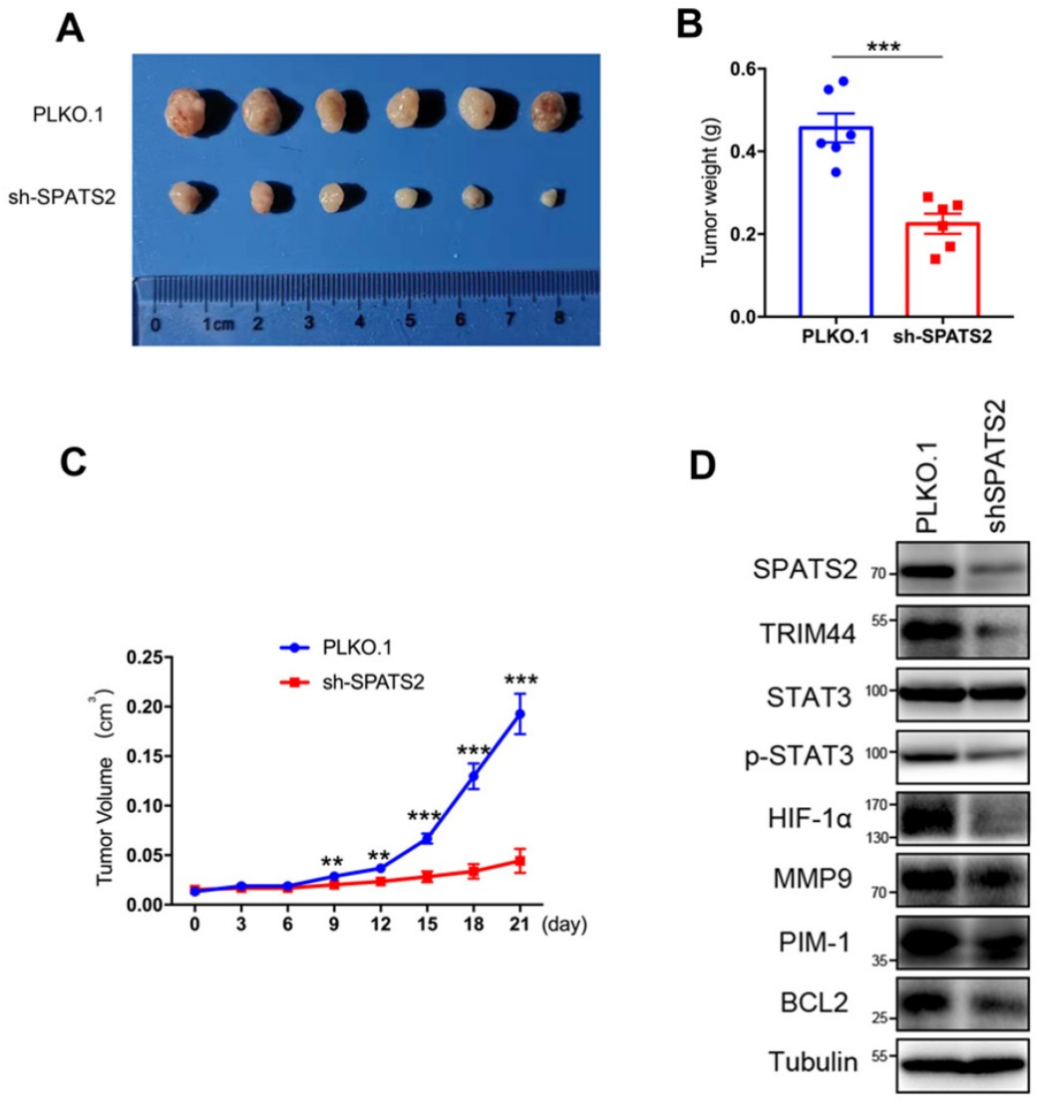
SPATS2 knockdown inhibits HCC tumor growth in vivo. (A) MHCC-97H (5×10^6^) were subcutaneously injected into balb/c mice (n=6). After 4 weeks, all mice were sacrificed, and tumors were dissected and imaged. (B) Tumor volume were measured using a caliper every three days after tumors were measurable (generally 7 days after MHCC-97H injection). (C) Tumors were weighed after dissection. (D) SPATS2, TRIM44, STAT3, p-STAT3 and their downstream protein were detected in tumor tissues by western blotting. Data were represented as mean ± SEM, *P < 0.05, **P <0.01, ***P < 0.001, vs. the marked control.

To investigate the effect of SPATS2 on the tumorigenic capacity of HCC cells in vivo, xenograft tumor growth assay was established by subcutaneous transplantation with either HCC-LM3- PLKO.1 or HCC-LM3-shSPATS2 cells into nude mice. The representative tumor images of two different groups were shown in Figure 3A. Consistent with results in vitro, silencing of SPATS2 strikingly reduced tumor size and weight compared to the control group (Figure 3B and C). Western blotting also confirmed that TRIM44, p-STAT3, BCL2, MMP9, HIF-1α and PIM1 was reduced with lower expression of SPATS2 in HCC-LM3-shSPATS2 xenograft tumors (Figure 3D). Collectively, depletion of SPATS2 diminishes xenograft tumor growth potential in vivo.

SPATS2 is upregulated in HCC and correlated with clinical outcomes.

**Figure 4:**
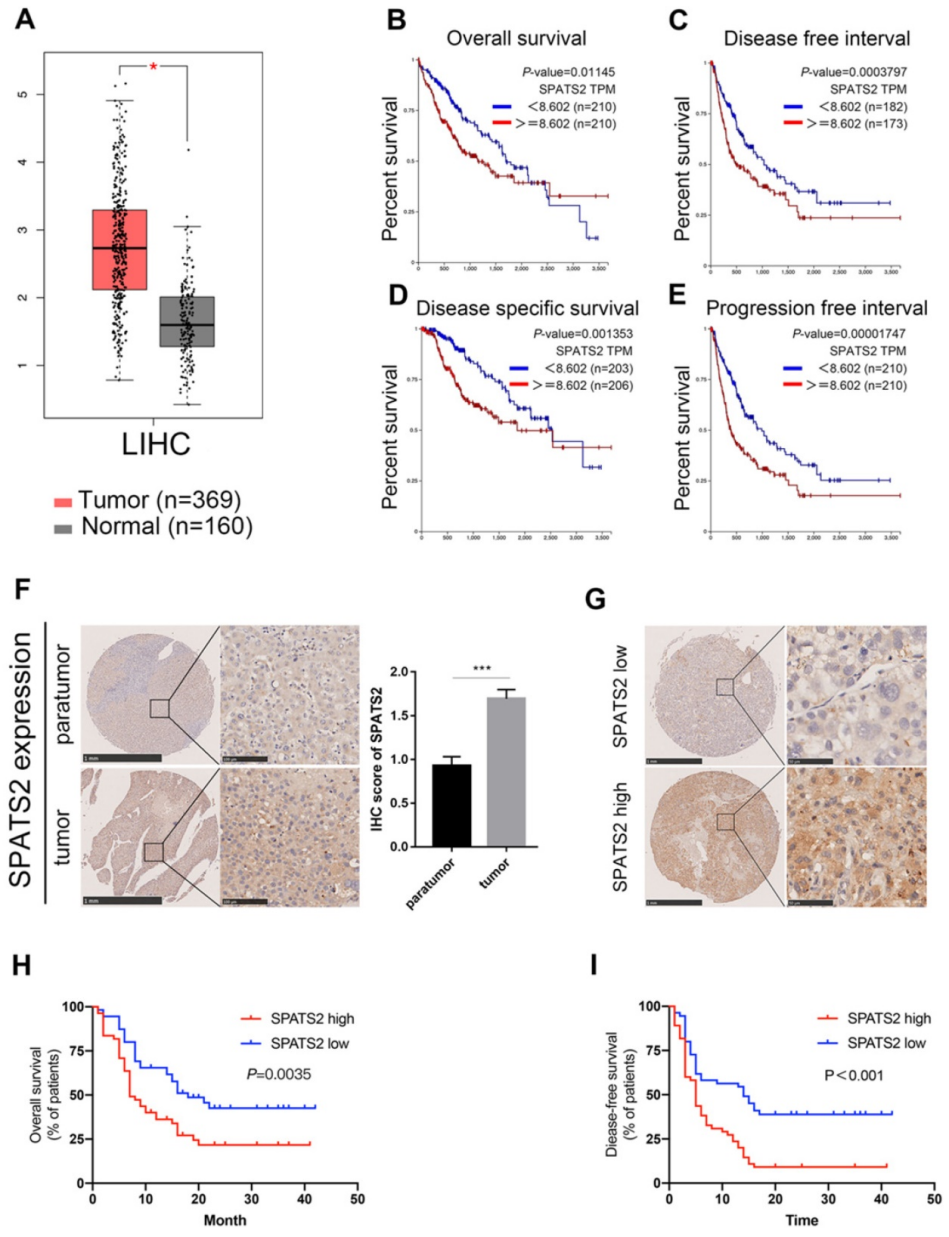
SPATS2 upregulates and drives poor prognosis in human HCC. (A) The expression of SPATS2 in LIHC and paired normal tissues by box plot. |Log2FC| Cutoff=1 and log2(TPM + 1) was used for log-scale. N=529. (B-E) Kaplan-Meier Curves for (B) overall survival(N=420), (C) disease free interval(N=355), (D) progression free interval(N=420) and (E) disease specific survival(N=409). (F) Immunohistochemical analysis of SPATS2 expression in HCC tissue microarrays. (G) Representative IHC images for SPATS2 expression with different staining intensity in HCC tissues. (H, I) Kaplan-Meier curves for (H) overall survival and (I) disease-free survival based on IHC scores of SPATS2 in an independent HCC cohort (n = 112). Log-rank test was used to compare the curve between patients with low and high expression level of SPATS2.The Y-axis represents survival probability, and the X-axis represents survival time (days/months). Data were represented as mean ± SEM, *P < 0.05, ***P < 0.001, vs. the marked control.

To clarify the role of SPATS2 in HCC, we explored its expression profile and clinical relevance based on the well-established TCGA database using GEPIA online tool (http://gepia.cancer-pku.cn/index.html) and UCSC Xena platform (https://xena.ucsc.edu/)^15^,^16^. Compared with normal tissues, SPATS2 expression was significantly higher in tumor specimens (Figure 4A). Furthermore, Kaplan-Meier analysis showed that higher SPATS2 expression was distinctly correlated with a reduction in overall survival, disease free interval, progression free interval and disease specific survival (Figure 4B-E).

To elucidate the clinical relevance in HCC patients, we measured SPATS2 protein expression level in 112 HCC tissue microarrays by immunohistochemical staining. A stronger staining intensity of SPATS2 in HCC tumor tissues was observed than that in para-tumor tissues (Figure 4F). Consistently, the IHC score of SPATS2 was remarkably higher in tumor tissues (Figure 4F). According to the SPATS2 staining intensity, we divided HCC patients into SPATS2-high and SPATS2-low groups (Figure 4G). Then, the correlation between SPATS2 expression and prognosis of HCC patients was investigated. Kaplan-Meier analysis revealed that high SPATS2 expression was significantly correlated with reduced overall survival (OS) and disease-free survival (DFS) in HCC patients (Figure 4H and I). As shown in Table 1, SPATS2 expression significantly correlated with sex (P=0.029), alanine aminotransferase (ALT) (P=0.001), maximal tumor size (P=0.005), microvascular invasion (P=0.007) and tumor differentiation (P<0.001).

The univariate analysis revealed that the ALT, AFP, maximal tumor size, microvascular invasion, tumor differentiation and SPATS2 expression were correlated with OS of HCC patients (Table 2). The AFP, maximal tumor size, microvascular invasion, tumor differentiation and SPATS2 expression were correlated with DFS of HCC patients (Table 3). Furthermore, multivariate Cox regression analysis showed that the SPATS2 expression could serve as an independent risk factor for both OS (Table 2) and DFS (Table 3) of HCC patients.

## Discussion

HCC is one of the most common malignant tumors worldwide with the clinical features of occult onset, rapid progression, early recurrence, and poor prognosis^17^. HCC is almost always advanced when clinically diagnosed^18^,^19^. By mining data from the integrated proteogenomic characterization of HBV related HCC, we identified that SPATS2 was upregulated and featured by increased proliferative proteins and decreased immune, inflammatory, and stromal proteins, which suggested SPATS2 might express highly in proliferative tumors^20^. Recently, SPATS2 gene expression level has been reported to be a prognostic marker in liver cancer, which lacked the information of how SPATS2 executed function in protein level and was far from enough to the association of SPATS2 with clinical characteristics 21. To our knowledge, no study has excavated the explicit role of SPATS2 in HCC. We hereby, for the first time, clarified the exact function and mechanism of SPATS2 in HCC.

To further validate the function of SPATS2 in HCC, we established SPATS2 knockdown HCC cell lines. Results of CCK‐8, colony formation assay, wound healing assay and transwell assay indicated that SPATS2 silencing could impede survival, proliferation, invasion and migration of HCC cells. In summary, our findings demonstrated that SPATS2 acted as an oncogene in HCC development.

Here we logically emphasized on excessive STAT3 activity in HCC and its association with SPATS2. The signal transducer and activator of transcription (STAT) proteins control a plethora of human malignancy processes^22^. The oncogenic functions of STAT3 have been extensively related to cancer cell proliferation, anti-apoptosis, migration, invasion, angiogenesis, stemness properties and immune suppression^23^. We reported that decreased phosphorylation of STAT3 in SPATS2 knockdown cells was detected along with downregulation of BCL2, MMP9, HIF-1α and PIM1. The change of BCL2 and PIM1 indicated the role of SPATS2-mediated p-STAT3 in HCC tumor growth, limitless replicative potential and resistance to apoptosis^24^,^25^. In terms of the digestion of extracellular matrices, downregulation of MMP-9 suggested that SPATS2 may induce migration and invasion via p-STAT3^26^. Moreover, decreased HIF-1α suggested the pro-angiogenic role of SPATS2-p-STAT3 axis in HCC, which is in accord with the marked vascularity characteristic of advanced HCC^27^. Thus, our data delineate a comprehensive SPATS2-mediated p-STAT3-associated biomarker profile, which mechanistically help define SPATS2 as an upstream molecule of STAT3.

TRIM44 plays a vital role in tumorigenesis and could serve as a prognostic biomarker and therapeutic target. In HCC, previous study showed that TRIM44 could strengthen resistance of HCC cells to doxorubicin by activating NF-κB^12^. In intrahepatic cholangiocarcinoma, elevated TRIM44 worsened the progression by inducing cell EMT via MAPK signaling^28^. In human esophageal cancer, TRIM44 was involved in the AKT/mTOR signaling pathway and STAT3 phosphorylation 10. TRIM44 was reported as a deubiquitinase to stabilize HIF-1α promoting survival of multiple myeloma cells in the osteoblastic niche^29^. In this study, we found that knockdown of SPATS2 could inhibit TRIM44 expression, thereby reducing the phosphorylation of STAT3 and then promoting the progression of HCC.

In addition, the in-vivo data provided support for positive correlation with SPATS2 and tumor growth. Consistently, the protein levels of TRIM44, p-STAT3, BCL2, MMP9, HIF-1α and PIM1 were all decreased in SPATS2 knockdown tumor tissues, which underpinned the contribution of SPATS2-TRIM44-p-STAT3 axis to tumorigenic events in HCC.

In this study, we analyzed SPATS2 gene expression in LIHC from the TCGA database. Significantly, SPATS2 was higher in tumor tissue than in normal tissue. Next, the above findings were consistent with our own cohort, which confirmed that high SPATS2 protein level is indicative of an unfavorable clinicopathological feature and poor prognosis and could serve as an independent risk factor for both OS and DFS of 112 HCC patients.

Taken together, we highlight the mechanism of SPATS2 in HCC and further investigations suggest the involvement of TRIM44-p-STAT3 signaling pathway mediated by SPATS2. The present study help elucidate the significance of SPATS2-TRIM44-p-STAT3 in HCC and provide a new theoretical basis for targeted therapy.

## Figures and Tables

**Table 1:** Correlation between SPATS2 expression and clinical characteristics

Variables		SPATS2 expression		F	*P*
		Low	High		
		(n=56)	(n=56)		
Sex	Female	6	15	4.869	**0.029**
	Male	50	41		
Age	≤50	25	19	0.000	0.246
	>50	31	37		
HBsAg	Negative	8	15	2.698	0.103
	Positive	48	41		
ALT	≤50U/L	42	25	11.662	**0.001**
	>50U/L	14	31		
AST	≤40U/L	20	25	0.920	0.340
	>40U/L	36	31		
AFP	≤10μg/L	19	11	2.938	0.088
	>10μg/L	37	45		
CEA	≤5μg/L	50	47	0.685	0.410
	>5μg/L	6	9		
CA19-9	≤40U/mL	37	35	0.153	0.696
	>40U/mL	19	21		
Cirrhosis	Absent	15	20	1.030	0.312
	Present	41	36		
Tumor number	Single	42	39	0.396	0.531
	Multiple	14	17		
Maximal tumor size	≤5cm	27	14	8.033	**0.011**
	>5cm	29	42		
Microvascular invasion	Absent	36	18	7.424	**0.001**
	Present	20	38		
Tumor differentiation	I-II	32	14	13.142	**0.001**
	III-IV	24	42		

**Table 2:** Univariate and multivariate analysis related with overall survival

	Univariate			Multivariate		
Variables	HR (95% CI)	*P*-value		HR	95% CI	*P*-value
Sex, male	1.142(0.645-2.02)	0.649				
Age ≥50, year	1.486(0.934-2.364)	0.094				
HBsAg, positive	0.807(0.442-1.474)	0.485				
ALT>50U/L	1.673(1.027-2.726)	**0.039**		2.234	(1.273-3.92)	**0.005**
AST>40U/L	1.404(0.881-2.238)	0.153				
AFP>10μg/L	0.33(0.173-0.628)	**0.001**				0.14
CEA>5μg/L	0.711(0.443-1.141)	0.158				
CA19-9>40U/mL	1.034(0.512-2.089)	0.925				
Cirrhosis, present	1.180(0.725-1.921)	0.505				
Tumor number, multiple	0.882(0.53-1.467)	0.629				
Maximal tumor size>5cm	0.272(0.156-0.475)	**<0.001**		0.387	(0.214-0.701)	**0.002**
Microvascular invasion, positive	0.388(0.236-0.637)	**<0.001**		0.527	(0.307-0.902)	**0.019**
Tumor differentiation, III-IV	0.596(0.37-0.961)	**0.034**		0.525	(0.323-0.885)	**0.01**
SPATS2 expression, high	0.518(0.323-0.831)	**0.006**		0.397	(0.226-0.696)	**0.01**

**Table 3:** Univariate and multivariate analysis related with disease-free survival

	Univariate			Multivariate		
Variables	HR (95% CI)	*P*-value		HR	95% CI	*P*-value
Sex, male	1.235(0.721-2.113)	0.442				
Age ≥50, year	1.242(0.802-1.924)	0.331				
HBsAg, positive	1.19(0.706-2.006)	0.514				
ALT>50U/L	1.213(0.78-1.887)	0.319				
AST>40U/L	1.241(0.803-1.916)	0.331				
AFP>10μg/L	0.404(0.233-0.7)	**0.001**				0.212
CEA>5μg/L	0.835(0.534-1.305)	0.429				
CA19-9>40U/mL	0.8(0.438-1.462)	0.469				
Cirrhosis, present	1.387(0.882-2.182)	0.157				
Tumor number, multiple	0.866(0.542-1.386)	0.549				
Maximal tumor size>5cm	0.358(0.22-0.584)	**<0.001**		0.423	(0.251-0.716)	**0.001**
microvascular invasion, positive	0.376(0.238-0.593)	**<0.001**		0.53	(0.327-0.858)	**0.01**
Tumor differentiation, III-IV	0.627(0.405-0.971)	**0.036**		0.464	(0.294-0.733)	**0.001**
SPATS2 expression, high	0.437(0.279-0.684)	**<0.001**		0.441	(0.274-0.771)	**0.001**

## Abbreviations

HCC: hepatocellular carcinoma
SPATS2: spermatogenesis associated serine rich 2
TRIM44: tripartite motif containing 44
STAT3: signal transducer and activator of transcription 3
ALT: alanine aminotransferase
RBPs: RNA-binding proteins
BCL2: BCL2 apoptosis regulator
MMP9: matrix metallopeptidase 9
HIF-1α: hypoxia inducible factor 1 subunit alpha
PIM1: pim-1 proto-oncogene, serine/threonine kinase
